# EML-SlowFast: A behavior recognition model for lion-head goose

**DOI:** 10.1016/j.psj.2025.105221

**Published:** 2025-05-02

**Authors:** Jinwei Wang, Zhiguo Du, Bin Wen, Zhihui Wu, Xudong Lin

**Affiliations:** College of Mathematics Informatics, South China Agricultural University, Guangzhou 510642, PR China

**Keywords:** Lion-head goose, behavior recognition, SlowFast, spatiotemporal features, computer vision in agriculture

## Abstract

The behavior of lion-head goose has a significant impact on their health status, activity levels, and productivity. It is therefore important to monitor the behavior of lion-head geese to enhance their health status, reproductive performance, and overall productivity. However, there is currently no specific behavioral recognition method for lion-head goose, which presents a significant challenge in quickly and effectively identifying various behaviors. To address this issue, this study proposes a model called EML-SlowFast, which is an improvement based on SlowFast. The model is capable of distinguishing five basic behaviors of lion-head goose: feeding, resting, preening, standing, and walking. The Efficient Channel Attention Bottleneck (**ECAbneck**) module and the Large Kernel Global-Local Feature Extraction (**LGLE**) module are designed and incorporated into the model. By combining and filtering channel information, the ECAbneck module enhances the model's ability to extract static characteristics from lion-head goose, increasing the accuracy of behavior recognition. The LGLE module captures temporal dependencies in lion-head goose behavior by integrating and extracting local and global features, thereby reinforcing the model's ability to model long-term temporal characteristics and further increasing accuracy. The experiment results showed that the average F1 score, average Precision, Accuracy, and average Recall of the EML-SlowFast model were 92.06 %, 91.60 %, 91.85 %, and 92.78 %, respectively, reflecting improvements of 4.03 %, 3.79 %, 4.14 %, and 4.45 % over the corresponding metrics of the SlowFast model. Furthermore, the FLOPs of the EML-SlowFast model was 10.807 G, which was a reduction of 7.358 G compared to the SlowFast model. Compared to commonly used behavior recognition models, the EML-SlowFast model has effective recognition of lion-head goose behaviors while maintaining low computational complexity, which is beneficial for deployment and use in scenarios with low computational resources. The EML-SlowFast model can rapidly and accurately recognize lion-head goose behaviors, providing a valuable reference for precision farming, reproduction, and health welfare monitoring of lion-head goose.

## Introduction

One of the biggest meat goose breeds worldwide, the Lion-head Goose is a germplasm resource that is specifically protected at the national level. It comes from Raoping in Chaozhou, Guangdong Province (Chen et al., 2011). It is characterized by its big size, great roughage tolerance, rapid growth rate, robust resilience to stress, and high-quality meat, which brings good economic benefits to breeders and traders (Lin et al., 2019a). In 2021, the lion-head goose industry chain generated a value of over 3.5 billion yuan, becoming a model for the goose industry in China. By May 2022, the annual production of lion-head geese in Shantou City reached about 8.1 million, with a stable breeding population of about 3 million (Luo et al., 2024). Although the industrialization of lion-head goose farming shows a rapid development trend, the application of the intensive farming model and new technologies such as "pen spraying" has also brought about challenges in disease control. At the current stage, although diseases can be prevented and controlled using antibiotics and vaccines, lion-head geese's health and productivity are negatively impacted by the irrational use of antibiotics and the limitations of vaccine specificity. (Liu et al., 2021). Lion-head geese are not weak in disease resistance, but due to problems such as weak investment capacity of farmers and poor rearing conditions, this leads to difficulties in epidemic prevention and control, and prominent biosafety issues (Luo et al., 2024).

The behavior of animals can typically serve as an indicator of their overall health and well-being, whether directly or indirectly (Qi et al., 2009). When animals are afflicted with disease, experience discomfort, or undergo physiological phases, they display distinctive behavioral traits (Lin et al., 2022b). Consequently, the monitoring of animal behavior can facilitate the early detection and prevention of animal diseases (Martiskainen et al., 2009; Barwick et al., 2018). The initial approach to monitoring animal behavior was based on manual observation. However, this approach was not only susceptible to subjective interpretation errors but also proved laborious and inefficient, rendering it unsuitable for the requirements of large-scale breeding. From the perspectives of profitability and animal welfare, a well-designed automatic monitoring and recognition classification system can effectively replace manual monitoring of animal behaviors (Stott et al., 2005). The implementation of Internet of Things (**IoT**)-based monitoring systems enables automated behavioral monitoring in animals via sensors or wearable devices (Adrion et al., 2018; Jin et al., 2024). However, such a contact-based method may cause issues such as animal stress and the potential for the devices to fall off or become damaged. The integration of computer vision with deep learning for the monitoring and analysis of animal behaviors has drawn a lot of academic interest due to the quick development of both fields. Computer vision-based methods for monitoring animal behavior offer several advantages, including non-contact, stress-free, and high-efficiency characteristics, which have led to their gradual replacement of wearable sensors in precision livestock farming (Su et al., 2022) and play an important role in the assessment of animal behaviors (Nasirahmadi et al., 2019).

Some scholars have implemented improved strategies including data augmentation, attention mechanisms, improved loss function, and improved feature fusion based on You Only Look Once (**YOLO**) object detection models to recognize poultry behaviors in single-frame images (Yang et al., 2024; Liu et al., 2023; Gu et al., 2022; Wang et al., 2024a; Xiao et al., 2023). However, single-frame-based methods for poultry behavior recognition can only extract spatial features from static images, which overlook the continuity and temporal characteristics of behavior (i.e., motion information). The analysis of poultry behavior is dependent on the recognition of a series of continuous activities. The temporal information between consecutive frames is of particular importance in determining the category of behavior, particularly when differentiating between activities and non-activities such as walking and standing. However, defining or classifying these features based solely on static images may present challenges. Therefore, the integration of spatial-temporal features becomes essential to achieve accurate recognition of poultry behaviors.

To effectively utilize the temporal features of poultry behaviors, some scholars have adopted temporal sequence modeling approaches based on skeletal keypoints. Feature learning models are employed to extract poultry skeletal keypoints from continuous single-frame images, followed by sequential modeling using models like Long Short-Term Memory **(LSTM**) to learn the temporal dependencies between keypoints across different poses (Fang et al., 2021; Nasiri et al., 2022; Volkmann et al., 2022). However, this methodology requires precise body and limb annotations for keypoint localization, incurring substantial data labeling costs.

Video understanding models demonstrate the capability to extract both the spatial and temporal features from videos simultaneously. Hu et al. (2024) proposed an end-to-end method called the Broiler Behavioral Recognition System (**BBRS**), which integrates an improved YOLOv8s detector, a ByteTrack tracker, and a 3D-ResNet50-TSAM model. The 3D-ResNet50-TSAM model is capable of simultaneously extracting spatial and temporal features of broiler behaviors from videos. The BBRS identifies five basic daily behaviors of broilers in videos with an overall accuracy of 93.98 %. Lin et al. (2022a) proposed a dual feature-rates deep fusion convolutional neural network **(DF2-Net**) incorporating a transposed non-local module for video-based bird behavior recognition. Through multi-rate feature integration and spatiotemporal relationship modeling, DF2-Net achieves an overall accuracy of 81.35 % in identifying eight avian behaviors (fighting, perching, feeding, swimming, flying, walking, standing, and eating).

In summary, research on computer vision-based poultry behavior recognition has achieved notable progress. Nevertheless, the methods employed for the recognition of lion-head goose behavior are still in their infancy. To bridge this gap, this study proposes EML-SlowFast model, which is based on the improved SlowFast, for recognizing the behavior of lion-head goose in videos. By integrating ECAbneck module and LGLE module into SlowFast, the EML-SlowFast model enables robust identification of five behaviors of the lion-head goose: feeding, resting, preening, standing, and walking.

## Materials and methods

### Materials

**Fig. 1:**
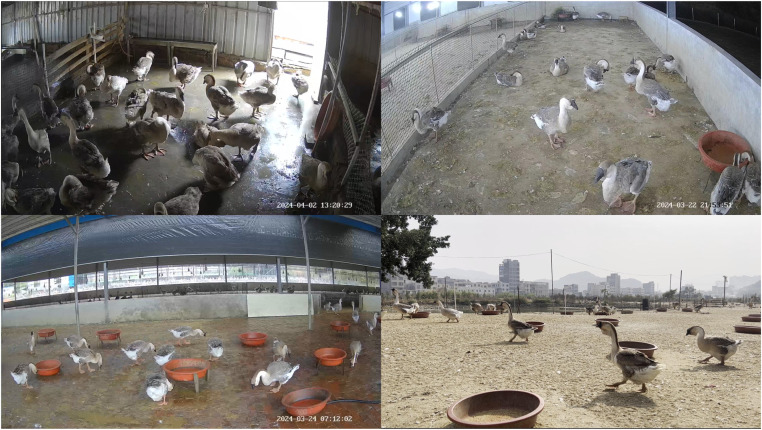
The samples of Lion-head Goose farming environments.

***Data acquisition*** The videos of lion-head geese were collected at two lion-head geese farms in Raoping, Chaozhou, Guangdong Province. The videos were collected from March 18, 2024, to July 12, 2024, at various times of the day, including mornings, afternoons, and evenings. The collection equipment consisted of multiple EZVIZ CS-C8b surveillance cameras. The videos were stored in MP4 format, and the camera resolution was 2304 × 1296 pixels with a frame rate of 15 FPS. The videos were collected from multiple different actual farming environments, as shown in Figure 1, enhancing the diversity of the data. The filming scenes included shelters and outdoor activity environments. In the shelter scenes, cameras were placed at the edges of the pens to ensure that the cameras could capture the lion-head geese's activity ranges as comprehensively as possible. The height varied according to the size of different pens. In outdoor activity scenes, cameras were placed in areas where the lion-head geese were frequently active. This study selected 191 usable videos from all the videos, with video durations ranging from 5 minutes to 20 minutes. These video data included various behaviors of lion-head goose.

**Fig. 2:**
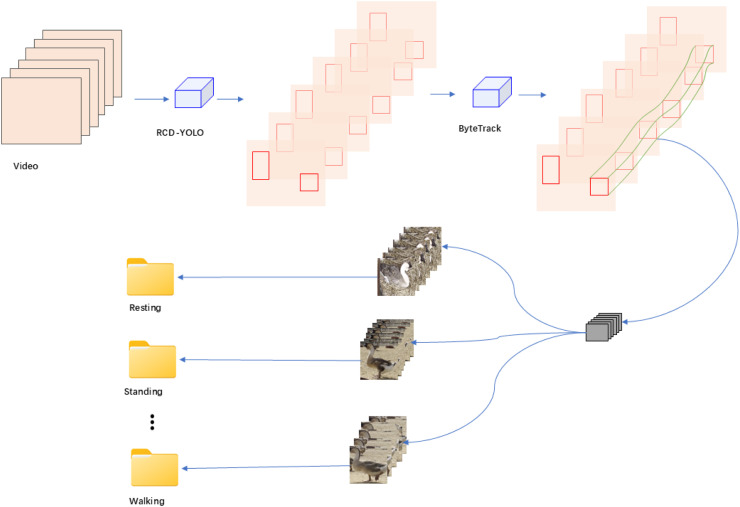
Dataset production flowchart.

**Fig. 3:**
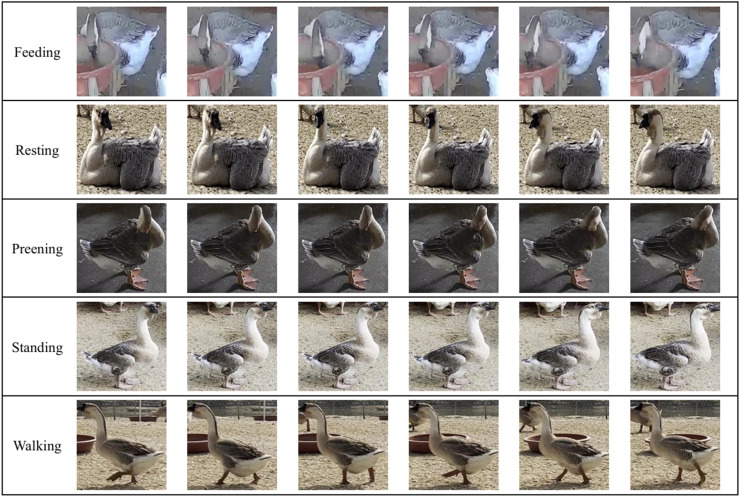
Examples of lion-head goose behaviors.

***Dataset construction*** The dataset was constructed in accordance with the format of the public dataset UCF101 (Soomro et al., 2012). The production process is shown in Figure 2. First, the video was input into the well-trained RCD-YOLO (Wang et al., 2024b) lion-head geese object detection network, which is responsible for detecting the lion-head geese objects, followed by the tracking of the lion-head geese using the ByteTrack algorithm (Zhang et al., 2022). Then, the detected lion-head geese that belonged to the same object ID in the image frame sequence were cropped and saved. This achieved the purpose of containing only one object in a single piece of data, which was beneficial for the subsequent behavior recognition model training. Next, the lion-head goose data were manually classified by behavior, and the image frame sequences corresponding to each behavior were saved as an instance of data into the folder corresponding to that behavior. The dataset contains five different behaviors, as shown in Figure 3.

After processing, a total of 2,966 pieces of data for five behaviors—feeding, resting, preening, standing, and walking—were obtained, with each piece of data containing the image sequence of 32 to 256 frames. The data were divided into a training set, a validation set, and a test set. Due to the varying activity levels of lion-head goose behavior, the distribution of lion-head goose behavior data was unbalanced, with the most data for standing behavior and the least for resting behavior. To achieve a balanced data distribution, data augmentation methods such as horizontal flip, vertical flip, invert color, Gaussian blur, and pepper noise were used to augment the training set. The specific dataset division is shown in Table 1.

**Table 1:** Lion-head goose behaviors dataset information.

behavior	Number of instances of data			
	Training		Validation	Test
	original	after augmentation		
Feeding	301	400	121	135
Resting	252	402	82	99
Preening	327	407	132	145
Standing	407	407	181	195
Walking	323	403	116	150

### Methods

***Overall*** In this research, EML-SlowFast, which was constructed based on the SlowFast architecture, the Efficient Channel Attention Bottleneck (ECAbneck) module, and the Large Kernel Global-Local Feature Extraction (LGLE) module, had been designed to recognize lion-head goose behaviors efficiently. EML-SlowFast is a lightweight and accurate lion-head goose behavior recognition model that introduces the ECAbneck module and LGLE module to improve static and dynamic feature extraction from lion-head goose behaviors. The EML-SlowFast effectively extracts both temporal and spatial features by considering the spatiotemporal features of video data, resulting in promising results in recognizing lion-head goose behaviors.

***SlowFast*** SlowFast (Feichtenhofer et al., 2019) is a 3D Convolutional Neural Network (**3DCNN**) for behavior recognition, with its architectural design influenced by the structural organization of the primate retina. In primates, approximately 80 % of retinal cells operate at a lower frequency for detailed spatial and color information, while the remaining 20 % operate at a higher frequency to capture motion changes. The SlowFast network comprises a Slow pathway designed to capture spatial semantics at a low frame rate, and a Fast pathway that efficiently captures rapidly evolving motion features at a high frame rate.

**Fig. 4:**
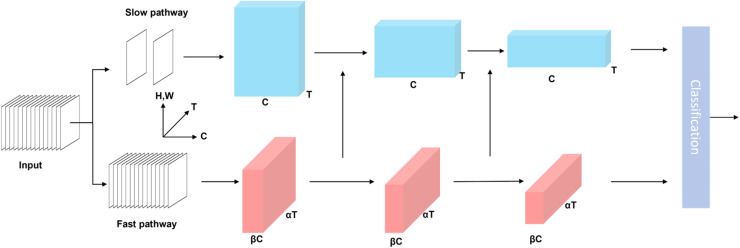
The structure of SlowFast.

The structure of SlowFast is illustrated in Figure 4. For the SlowFast network, with an input sequence length of t frames, the Slow pathway samples $T\;=\frac{t}{\tau }\;$frames with a large τ (often 16), capturing spatial details. The Fast pathway samples αT frames with $\frac{\tau }{\alpha }$ (often α = 8), capturing motion details. To balance computation, the Fast pathway has β times fewer channels than the Slow pathway (β is often $\frac{1}{8}$). The Fast and Slow pathways employ 3D ResNet blocks (Hara et al., 2018) as their backbone for spatiotemporal feature extraction. Additionally, through lateral connections, features extracted from the Fast pathway are fused into the Slow pathway, enhancing the interaction between static and dynamic behavioral features. Ultimately, the features extracted from both pathways are merged and utilized for prediction to produce classification results.

**Fig. 5:**
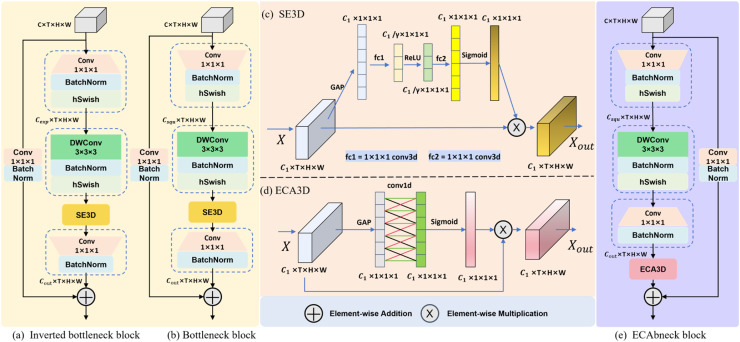
Lightweight feature extraction module.

***Lightweight feature extraction module*** The backbone of the Slow pathway in SlowFast is designed to extract static features using 3D ResNet blocks, which are inefficient and computationally complex. The Inverted Bottleneck (**IB**) block, originating from MobileNetV3 (Howard et al., 2019), is a lightweight 2D module. In this study, the IB block has been extended to a 3D module, as illustrated in Figure 5(a). The IB block employs an inverted bottleneck structure, which first expands the number of channels for the input feature map to $C_{exp}$ using a 1 × 1 × 1 convolution. This expansion allows the feature map to contain more high-dimensional information. Subsequently, a depth-wise convolution (**DWConv**) is applied for the purpose of feature extraction. Subsequently, a Squeeze-and-Excitation (**SE**) module (Hu et al., 2018) is utilized, which is a channel attention mechanism that assigns different levels of importance to channels by compressing and then expanding the channel dimension through a squeeze-excitation process. DWConv is a lightweight convolution that reduces model complexity; however, it lacks inter-channel information interaction. So, after the SE module, a 1 × 1 × 1 convolution is immediately applied, serving two purposes: firstly, to facilitate inter-channel information interaction and secondly, to adjust the number of feature map channels to the output channels ($C_{out}$). Finally, a shortcut connection is employed, which is a 1 × 1 × 1 convolutional layer used to adjust the number of channels in the feature map, adding the input feature map to the output feature map.

In SlowFast, due to the large number of channels for the feature maps within the Slow pathway, expanding the channels would significantly increase the computational complexity of the model. Therefore, we modified the IB block into a Bottleneck (**bneck**) block by altering the inverted bottleneck structure to the bottleneck structure, as shown in Figure 5(b). The compression of the number of input channels allows for the integration of high-dimensional channel information, enabling more efficient feature extraction. We used the bneck blocks to replace the 3D ResNet blocks within the Slow pathway, effectively reducing the complexity of the model. However, the model parameters increased significantly, and experiments revealed that this was due to the SE module. To address this issue, we improved the bneck module and designed a more efficient module called Efficient Channel Attention Bottleneck (**ECAbneck**), whose structure is illustrated in Figure 5(e). Firstly, the SE module is replaced with the Efficient Channel Attention (**ECA**) module (Wang et al., 2020) to learn the channel information and enhance the discriminability of features. The ECA module is an efficient channel attention; unlike the SE module, which employs 3D convolution for squeeze-excitation operations, the ECA module solely utilizes a 1D convolution to learn the information of channel dimension, thereby significantly reducing model parameters. The ECA module obtains the aggregated features by global average pooling (**GAP**), followed by generating channel weights by performing a 1D convolution, and then applying the channel weights to the input feature map to obtain the channel attention. This process can be computed by Equations (1)-(2):

(1) $$X^{\prime }=\sigma \left(Conv1d\left(GAP\left(X\right)\right)\right)$$

(2) $$X_{out}=X^{\prime }⊗X$$

where X is the input feature map of the ECA module, $X\in R^{C\times T\times H\times W}$, GAP denotes global average pooling, Conv1d is a 1D convolution, σdenotes sigmoid activation function, ⊗ denotes element-wise multiplication operation.

Subsequently, the position of the channel attention module and the last 1 × 1 × 1 convolution layer within the bneck block is swapped. The rationale behind this adjustment is that the channels contain richer information and are more interconnected in the 3D feature map. After feature extraction using DWConv, immediately applying a 1 × 1 × 1 convolution layer for inter-channel information interaction can strengthen the association between channels. Following this, applying the attention module to learn channel weights allows for a more effective extraction of the channel features.

In this study, the ECAbneck modules were used to replace the 3D ResNet blocks within the Slow pathway, effectively reducing the computational complexity of the model. By employing the bottleneck structure along with the ECA module, the extraction capability of the Slow pathway for static behavioral features was augmented, which subsequently enhanced the model's accuracy in recognizing the behaviors of lion-head goose.

**Fig. 6:**
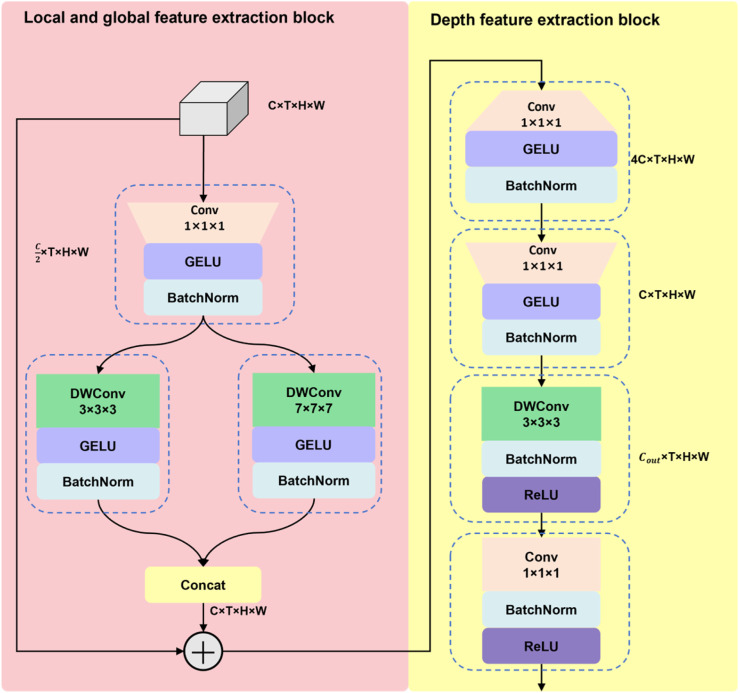
The structure of the LGLE module.

***Large Kernel Global-Local Feature Extraction module*** The Fast pathway of SlowFast has fewer channels and more temporal dimensions, primarily responsible for the extraction of dynamic features. We designed the Large Kernel Global-Local Feature Extraction (**LGLE**) module, which is comprised of a local and global feature extraction block, along with a depth feature extraction block. The structure of the LGLE module is shown in Figure 6. The LGLE module enhances the model's long-term modeling capability and depth feature extraction capability, so that the model can better mine the semantic information of the lion-head goose's behavior and improve the model's ability to model the long-term behavioral characteristics of the lion-head goose.

**Local and global feature extraction block.** Firstly, the input feature map is processed through a 1 × 1 × 1 convolution layer, which squeezes the number of channels for the input feature map. The channel squeezing operation not only reduces the computational load by decreasing the number of channels but also facilitates the interaction of channel information, thereby benefiting the model's extraction of behavioral semantics from the lion-head goose. Subsequently, the feature map undergoes parallel processing with a 3 × 3 × 3 DWConv layer and a 7 × 7 × 7 DWConv layer. The 3 × 3 × 3 DWConv layer is employed for extracting local features, focusing on the detailed information within the lion-head goose's behavior, whereas the 7 × 7 × 7 DWConv layer is employed for extracting global features, thereby enhancing the module's long-term modeling capability. This enables the module to capture the correlations between information across longer temporal channels, which is beneficial for the capability of the model to learn the temporality of the lion-head goose's behavior. Subsequently, the local and global feature maps are fused and then subjected to a residual connection with the input feature map, after which the result is outputted. The expression of the local and global feature extraction block is shown in Equations (3)-(6).

(3) $$\begin{array}{c} F^{\text{'}}=\mathrm{BN}\left(\sigma_{1}\left\{\mathrm{Con}v_{1\times 1\times 1}\left(F\right)\right\}\right) \end{array}$$

(4) $${F^{\prime }}_{local}=BN\left(\sigma_{1}\left\{DWConv_{3\times 3\times 3}\left(F^{\prime }\right)\right\}\right)$$

(5) $${F^{\prime }}_{global}=BN\left(\sigma_{1}\left\{DWConv_{7\times 7\times 7}\left(F^{\prime }\right)\right\}\right)$$

(6) $$F^{\prime \prime }=Concat\left({F^{\prime }}_{{}_{local}},{F^{\prime }}_{global}\right)+F$$

where F is the input feature, $F\in R^{C\times T\times H\times W}$, Conv and DWConv denote convolutional layer and DWConv layer, respectively, and the subscript represents the size of the convolutional kernel, $\sigma_{1}$ denotes the GeLU activation function, BN denotes the Batch Normalization layer, Concat denotes the concat layer, and $F^{\prime \prime }$ is the output feature map for the local and global feature extraction block.

**Depth feature extraction block.** The output of the local and global feature extraction block serves as the input feature map for the depth feature extraction block. Initially, it passes through a 1 × 1 × 1 convolutional layer to expand the number of channels by fourfold, and then the number of channels is restored through another 1 × 1 × 1 convolutional layer. The "expand-and-compress" operation augments the interaction between channels, thereby facilitating a deeper integration of features within the feature map. Subsequently, a 3 × 3 × 3 DWConv layer and a 1 × 1 × 1 convolutional layer are applied to the feature map for further feature learning. The expression of the depth feature extraction block is shown in Equations (7)-(10).

(7) $$F_{\exp}^{\prime \prime }=BN\left(\sigma_{1}\left\{Conv_{1\times 1\times 1}\left(F^{\prime \prime }\right)\right\}\right)$$

(8) $$F_{comp}^{\prime \prime }=BN\left(\sigma_{1}\left\{Conv_{1\times 1\times 1}\left(F_{\exp}^{\prime \prime }\right)\right\}\right)$$

(9) $$F_{d}^{\prime \prime }=\sigma_{2}\left\{BN\left(DWConv_{3\times 3\times 3}\left(F_{comp}^{\prime \prime }\right)\right)\right\}$$

(10) $$F_{out}=\sigma_{2}\left\{BN\left(Conv_{1\times 1\times 1}\left(F_{d}^{\prime \prime }\right)\right)\right\}$$

where $\sigma_{2}$ denotes the ReLU activation function, $F_{out}$ is the output feature map of the depth feature extraction block and is also the output feature map of the LGLE module.

The local and global feature extraction block employs a 3 × 3 × 3 DWConv layer and a 7 × 7 × 7 DWConv layer to capture both local and global features, which are beneficial for long-term temporal modeling. This effectively enhances the module's capacity to model the temporality of lion-head goose behavior. The depth feature extraction block fuses the feature map in a deep manner, thereby enabling the module to more effectively learn the spatial and temporal characteristics of lion-head goose behavior. In this study, the LGLE modules, consisting of the local and global feature extraction block and the depth feature extraction block, replace the 3D ResNet blocks within the Fast pathway of SlowFast network, effectively enhancing the model's long-term modeling capability. This modification strengthens the model's proficiency in learning the spatial and temporal features of lion-head goose behavior, ultimately improving the model's performance in classifying lion-head goose behavior.

**Fig. 7:**
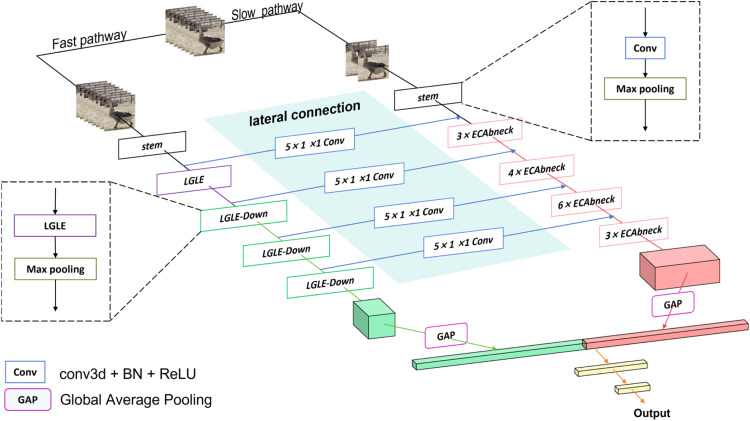
The structure of EML-SlowFast network.

***EML-SlowFast Behavior Recognition Model*** In this study, the EML-SlowFast behavior recognition model based on SlowFast improvement was designed for recognizing the behavior of lion-head goose. The structure of the EML-SlowFast network is shown in Figure 7. Firstly, to enhance the static behavioral feature extraction capacity of the Slow pathway, the 3D ResNet blocks within the Slow pathway of SlowFast were replaced with the ECAbneck modules. The ECAbneck module establishes dependencies between spatiotemporal information and channel features, thereby generating more discriminative feature representations through the fusion of richer information. Secondly, the innovative LGLE modules were implemented as the replacement for the 3D ResNet blocks within the Fast pathway of SlowFast. The LGLE module incorporates the principles of large kernel convolution and bottleneck structures, enabling the effective extraction of both local and global features while enhancing the model's capacity for long-term spatiotemporal modeling. This enables the model to more effectively extract information from the temporal channels, thereby enhancing its ability to extract dynamic behavioral features in the temporal dimension. Concurrently, between the Slow and Fast pathways, convolutional layers were employed as lateral connections to fuse features extracted by the Fast pathway into the Slow pathway, thus enhancing the interaction between static and dynamic behavioral features.

**Fig. 8:**
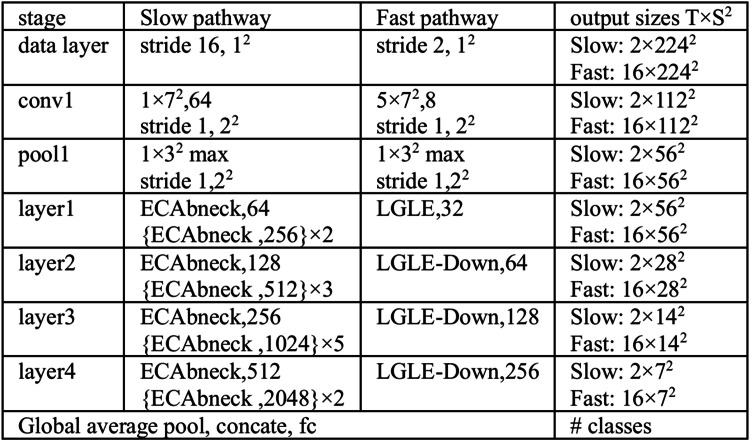
The instantiation of the EML-SlowFast network. The dimensions of kernels are denoted by {*T* × S^2^, C} for temporal, spatial, and channel sizes. Strides are denoted as {temporal stride, spatial stride^2^}.

The specific network information of the EML-SlowFast is shown in Figure 8. The values of τ, α, and β have been set to 16, 8, and $\frac{1}{8}$, respectively. The input data is a 32-frame image sequence. In the Slow pathway, data is sampled at a low rate to obtain a 2-frame image sequence, while in the Fast pathway, data is sampled at a high rate to obtain a 16-frame image sequence. In the Slow pathway, the data initially undergoes feature extraction through a 3D convolution layer with a kernel size of 1 × 7 × 7 and a stride of 1 × 2 × 2, followed by max pooling for downsampling to obtain early feature maps. Subsequently, it passes through consecutive ECAbneck modules for feature extraction, and fusion with the feature maps from lateral connections, employing 3D convolution layers with a kernel size of 5 × 1 × 1, to obtain the output feature maps of the Slow pathway. In the Fast pathway, the data initially undergoes feature extraction through a 3D convolution layer with a kernel size of 5 × 7 × 7 and a stride of 1 × 2 × 2, followed by max pooling for downsampling to obtain an early feature map. Subsequently, the feature map passes through an LGLE module and three LGLE-Down modules to obtain the output feature map of the Fast pathway. Finally, the output feature map from both the Slow and Fast pathways undergoes a global average pooling, after which the features are then fused and input into a fully connected (**fc**) layer for classification.

### Experimental platform and parameters setting

The experiments in our study were conducted on a computer equipped with an Intel Xeon Platinum 8481C CPU and an NVIDIA GeForce RTX 4090D GPU, running the Ubuntu 22.04 operating system. The network environment was a virtual environment created using Anaconda3, which included Python 3.10, PyTorch 2.1.0, and CUDA 12.1. The training parameters for the experiments are presented in Table 2.

**Table 2:** Training parameter settings.

Parameters	Value
Size of input image	224 × 224
Batch size	16
Epochs	300
Optimizer	Stochastic Gradient Descent (SGD)
Initial learning rate	0.05
Momentum	0.9
Weight decay	0.0001

### Evaluation metrics

To evaluate the recognition performance of the proposed model for different behaviors of lion-head goose in this study, Accuracy, Parameters, Precision, Recall, F1 score, and Floating Point Operations per second (**FLOPs**) are used as the performance evaluation metrics. Specifically, Precision is defined as the ratio of correctly predicted positive cases to all predicted positive cases. Recall is defined as the ratio of correctly predicted positive cases to all positive cases. Accuracy is the average of the precision rates for all behaviors. FLOPs and Parameters are used to measure the computational and spatial complexity of the network, respectively. The F1 score combines the discriminative capabilities of Precision and Recall to assess the model's overall performance. The calculations for Precision, Recall, Accuracy, and F1 score are shown in Equations (11)–(14).

(11) $$\text{Precision}=\frac{TP}{TP+FP}$$

(12) $$\text{Recall}=\frac{TP}{TP+FN}$$

(13) $$\text{Accuracy}=\frac{TP+TN}{TP+TN+FP+FN}$$

(14) $$F1=2\times \frac{\text{Precision}\times \text{Recall}}{\text{Precision}+\text{Recall}}$$

where TP, FP, FN, and TN represent the number of true positive cases, false positive cases, false negative cases, and true negative cases, respectively.

## RESULTS AND DISCUSSION

### The Effect of EML-SlowFast on Behavior Recognition in Lion-head Goose

**Fig. 9:**
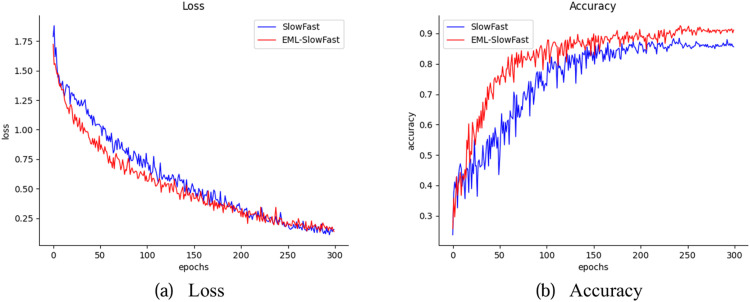
(a) Model loss comparison, (b) Model accuracy comparison.

Figure 9 shows the loss and accuracy curves of the training process for SlowFast and EML-SlowFast. It can be observed that the loss curve of EML-SlowFast decreases earlier, and the accuracy curve of EML-SlowFast rises more rapidly, suggesting that the EML-SlowFast model approaches a state of fitting more swiftly.

To validate the effectiveness of the improvement in this study, we conducted ablation experiments using the SlowFast network with backbone 3DResNet50 as the baseline. Table 3 displays the results of these ablation experiments. Following the replacement of the 3D ResNet blocks within the Slow pathway with the bneck modules an increase of 0.96 % in Accuracy was observed, accompanied by a decrease of 9.128 G in FLOPs. However, the network's parameters increased by 46.216 M, due to redundancy in the SE module within the bneck module. Further, the replacement of the 3D ResNet blocks within the Slow pathway with the ECAbneck modules resulted in a notable reduction in the network's parameters, when compared to the "SlowFast + bneck" method. This indicates that the ECA module, compared to the SE module, eliminates a considerable amount of parameter redundancy. Nonetheless, the Accuracy of "SlowFast + ECAbneck" method decreased by 1.24 % compared to "SlowFast + bneck" method. This is since while the ECA module enhances the Slow pathway's capacity to extract static features, it does not equally augment the Fast pathway's capacity to extract dynamic features. This leads the network to overly focus on static features, reducing the emphasis on dynamic features, thus hindering its effectiveness in capturing the temporal relationships of behavior. To explore the effectiveness of the LGLE module, the 3D ResNet blocks within the Fast pathway of SlowFast were replaced with the LGLE modules. Compared to the baseline, Accuracy increased by 2.07 % and the number of parameters of the model decreased by 0.174 M. This shows that the LGLE module effectively enhances the network's long-term modeling capabilities, enabling it to effectively capture the temporal relationships of behavior. For the "SlowFast + bneck + LGLE" method, replacing the 3D ResNet blocks in both the Slow and Fast paths with bneck modules and LGLE modules, respectively, resulted in a 2.76 % increase in Accuracy and a reduction of 7.348 G FLOPs compared to the baseline. This is due to the lower FLOPs of the bneck module, and its channel attention makes the network focus on important features, thus both reducing network computational complexity and improving static feature extraction for lion-head goose behavior. The LGLE block combines and learns from both local and global features, enabling long-term modeling capability that facilitates the network's ability to capture the temporal nature of the lion-head goose's behavior, thereby strengthening the model's capacity for dynamic feature extraction. However, this method comes with a relatively high number of parameters. Further, by replacing the bneck modules with the ECAbneck modules, the model achieved a 4.14 % increase in Accuracy, reaching 91.85 %, and a reduction of 7.358 G FLOPs, compared to the baseline. Although the number of parameters increased by 5.727 M, this increase falls within an acceptable range. The above results show that the ECAbneck module and the LGLE module for the improvement of SlowFast in this study can increase the accuracy and reduce FLOPs in the model.

**Table 3:** Contribution of different modules to the EML-SlowFast model.

bneck	ECAbneck	LGLE	#Params(M)	FLOPs(G)	Accuracy (%)
×	×	×	33.571	18.165	87.71
**√**	×	×	79.787	9.037	88.67
×	**√**	×	39.472	9.027	87.43
×	×	√	33.397	19.945	89.78
√	×	√	79.612	10.817	90.47
×	√	√	39.298	**10.807**	**91.85**

To validate the recognition performance of EML-SlowFast, an analysis of the confusion matrices obtained from experiments conducted on the test set for both SlowFast and EML-SlowFast is presented, with the results summarized in Table 4. The results indicated that the Precision, Recall, and F1 score of EML-SlowFast are greater than those of SlowFast, except for the Recall of walking behavior. The EML-SlowFast model achieved the F1 score of 96.68 %, 90.57 %, 91.22 %, 87.94 %, and 93.92 % for the classification of feeding, resting, preening, standing, and walking behaviors, which were higher than those of SlowFast model by 2.26 %, 4.57 %, 5.7 %, 6.56 % and 1.11 %, respectively. In terms of classification performance for walking behavior, the EML-SlowFast model achieved a Precision that was 4.18 % higher than that of the SlowFast model, but its Recall was lower than that of the SlowFast model by 2 %. This may be attributed to the enhanced feature discrimination capability of the EML-SlowFast model through integration of the ECAbneck and LGLE modules. This improvement leads to stricter classification criteria, causing partially ambiguous samples (e.g., instances with occluded leg regions of the goose where only partial motion patterns are observable in walking sequences) to be misclassified as negative cases, which explains the observed 2 % Recall decrease. Nonetheless, the EML-SlowFast model still achieved an impressive F1 score of 93.92 % for walking behavior classification, demonstrating superior comprehensive performance. Furthermore, for behaviors involving subtle movements such as feeding and preening, which rely on the model's static and dynamic feature extraction capability, the EML-SlowFast model exhibits even better performance. The EML-SlowFast model only exhibits lower Recall than SlowFast in the classification of walking behavior, while its performance in other classifications surpasses SlowFast. Furthermore, the average Recall, average Precision, and average F1 score of the EML-SlowFast model were 4.45 %, 3.79 %, and 4.03 % higher than those of SlowFast, respectively. This indicates that the overall performance of the EML-SlowFast model in recognizing the behaviors of lion-head goose is superior to that of SlowFast. Thus, the validity of the proposed model in this paper is confirmed.

**Table 4:** Confusion matrix analysis results.

Behavior	SlowFast			EML-SlowFast		
	Precision(%)	Recall(%)	F1(%)	Precision(%)	Recall(%)	F1(%)
Feeding	94.78	94.07	94.42	96.32	97.04	96.68
Resting	85.15	86.87	86.00	84.96	96.97	90.57
Preening	83.55	87.59	85.52	89.40	93.10	91.22
Standing	84.53	78.46	81.38	92.13	84.10	87.94
Walking	91.03	94.67	92.81	95.21	92.67	93.92
average	87.81	88.33	88.03	**91.60**	**92.78**	**92.06**

### Comparison with other behavior recognition models

To demonstrate the effectiveness of EML-SlowFast in recognizing the behavior of lion-head goose, it was compared with other commonly used convolutional networks in the field of video behavior recognition, including TSN, I3D, R(2 + 1)D, C2D and TSM, with the results presented in Table 5 (Wang et al., 2016; Carreira and Zisserman, 2017; Tran et al., 2018; Wang et al., 2018; Lin et al., 2019b). The EML-SlowFast model had Parameters, FLOPs, and Accuracy of 39.298 M, 10.807 G, and 91.85 %, respectively. Among all the comparative models, the EML-SlowFast model had the lowest FLOPs, and its Accuracy was only 0.28 % lower than that of the TSM model. Moreover, the FLOPs of the TSM model reached 42.939 G, which was 32.132 G higher than that of the EML-SlowFast model, making its deployment challenging in scenarios with limited computational resources. Achieving 91.85 % Accuracy with 10.807 G FLOPs, the EML-SlowFast model offers the best cost-performance ratio and is suitable for deployment in low computational resource scenarios. Overall, the EML-SlowFast model exhibits the best comprehensive performance, offers the highest cost-performance ratio, and can provide efficient and stable results for lion-head goose behavior recognition.

**Table 5:** Comparison of different behavior recognition models.

network	#Params(M)	FLOPs(G)	Accuracy (%)
I3D-3DResNet50	27.236	21.728	79.28
R(2 + 1)D-3DResNet34	63.557	106	83.70
C2D-ResNet50	23.52	50.788	79.83
TSN-ResNet50	23.52	42.939	87.43
TSM-ResNet50	23.52	42.939	92.13
SlowFast-3DResNet50	33.571	18.165	87.71
EML-SlowFast(**Our**)	39.298	10.807	91.85

### Analyzing the regions of interest of EML-SlowFast model for lion-head goose behavior recognition

**Fig. 10:**
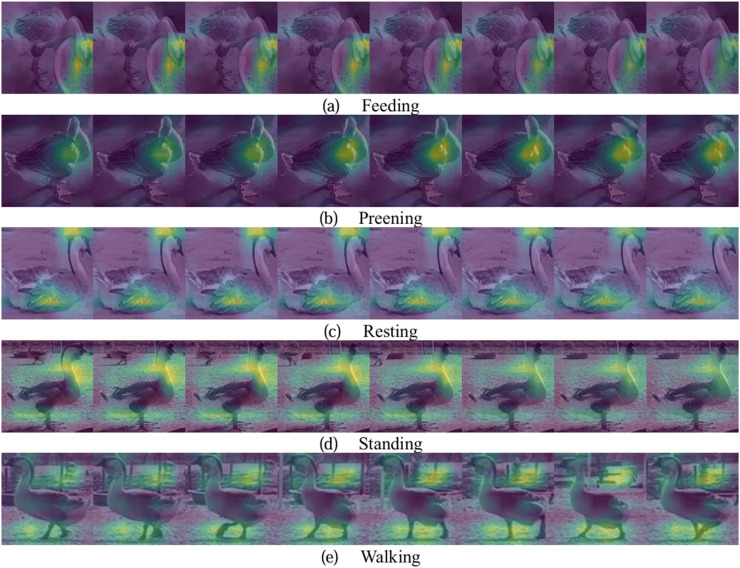
The regions of interest of the EML-SlowFast model visualization results of lion-head goose behaviors.

The Gradient-weighted Class Activation Mapping (**Grad-CAM**) technique (Selvaraju et al., 2017) was used to visualize the regions of interest of the EML-SlowFast model for different behaviors, as shown in Figure 10. The brightly colored regions highlight the areas of interest identified by the model, where the features correspond to the lion-head goose's behavior. For feeding behavior, the model focuses on the feeding trough and the lion-head goose's head. For preening behavior, the model focuses on the area where the lion-head goose preens its feathers. For resting behavior, the model focuses on the lion-head goose's body and head, where stationary body and possibly moving head contribute to identifying resting behavior. For standing behavior, the model focuses on the lion-head goose's feet and head, where stationary feet and a possibly moving head contribute to identifying standing behavior. For walking behavior, the model primarily focuses on the lion-head goose's feet and the background, where foot movement and background changes in a sequence of frames determine walking behavior. In summary, the model's regions of interest align with the characteristics of lion-head goose behaviors, confirming the EML-SlowFast model's validity in behavioral cognition.

## Conclusions

Automated recognition of lion-head goose behavior can effectively reduce human resources, achieve refined and intelligent supervision and management of breeding farms, and improve the health level of lion-head goose, enabling timely detection and isolation of abnormal individuals. However, there are currently no available methods for the recognition of lion-head goose behavior. To bridge this gap, this study proposes a model termed EML-SlowFast for the automatic and accurate recognition of five lion-head goose behaviors (feeding, resting, preening, standing, and walking) in videos. In addition, we constructed a lion-head goose behavior dataset containing 2,966 behavioral sequence samples (32 to 256 image frames per sequence) covering five fundamental lion-head goose behaviors: feeding (*n* = 557), resting (*n* = 433), preening (*n* = 604), standing (*n* = 783), and walking (*n* = 589). This dataset was developed for training and validating the EML-SlowFast model, while also serving as a foundational resource to facilitate future research in lion-head goose behavior analysis.

To achieve the recognition of lion-head goose behaviors, this study improved the SlowFast network and proposed EML-SlowFast model. The specific approach includes replacing the 3D ResNet blocks within the Slow pathway with the ECAbneck modules, which utilize the channel attention ECA to construct spatial-temporal information and inter-channel dependencies. By combining richer information, the ECAbneck module creates more unique feature illustrations, improving the model's ability to extract static features. Innovatively, the LGLE module had been designed and utilized to replace the 3DResNet blocks within the Fast pathway. The LGLE module integrates the concepts of large kernel convolution and bottleneck structures, effectively extracting local and global features, and strengthening the model's capacity to model long-term spatial-temporal features. This allows the model to more effectively extract features from the temporal channels, enhancing its ability to extract dynamic features. The experiment results demonstrated the improvement effects of the ECAbneck modules and the LGLE modules on the SlowFast network and the enhancement of recognition accuracy. The EML-SlowFast model achieved the average F1 score, average Precision, Accuracy, and average Recall of 92.06 %, 91.60 %, 91.85 %, and 92.78 %, respectively, reflecting improvements of 4.03 %, 3.79 %, 4.14 %, and 4.45 % over the corresponding metrics of the SlowFast model. Furthermore, the FLOPs of the EML-SlowFast model was 10.807 G, which was a reduction of 7.358 G compared to the SlowFast model. Compared to commonly used behavior recognition models, the EML-SlowFast model has effective recognition of lion-head goose behaviors while maintaining low computational complexity, which is beneficial for deployment and use in scenarios with low computational resources.

In summary, the EML-SlowFast model effectively and accurately performs the task of lion-head goose behavior recognition.

## Declaration of competing interest

The authors declare that they have no known competing financial interests or personal relationships that could have appeared to influence the work reported in this paper.

## References

[bib0001] Adrion F., Kapun A., Eckert F., Holland E.M., Staiger M., Götz S., Gallmann E. (2018). Monitoring trough visits of growing-finishing pigs with UHF-RFID. Comput. Electron. Agric..

[bib0002] Barwick J., Lamb D., Dobos R., Schneider D., Welch M., Trotter M. (2018). Predicting lameness in sheep activity using tri-axial acceleration signals. Animals.

[bib0003] Carreira J., Zisserman A. (2017). proceedings of the IEEE Conference on Computer Vision and Pattern Recognition.

[bib0004] Chen J., Peng X., Lu J., Sun J., Qiu J., Tang X., Zhang J. (2011). Anti-season farming technology of original lion head goose (in Chinese). Guangdong Agricult. Sci..

[bib0006] Fang C., Zhang T.M., Zheng H.K., Huang J.D., Cuan K.X. (2021). Pose estimation and behavior classification of broiler chickens based on deep neural networks. Comput. Electron. Agric..

[bib0007] Feichtenhofer C., Fan H., Malik J., He K. (2019). Proceedings of the IEEE/CVF international conference on computer vision.

[bib0008] Gu Y., Wang S., Yan Y., Tang S., Zhao S. (2022). Identification and analysis of emergency behavior of cage-reared laying ducks based on YoloV5. Agriculture.

[bib0009] Hara K., Kataoka H., Satoh Y. (2018). Proceedings of the IEEE conference on Computer Vision and Pattern Recognition.

[bib0010] Howard A., Sandler M., Chu G., Chen L.C., Chen B., Tan M., Wang W.J., Zhu Y.K., Pang R.M., Vasudevan V., Le Q.V., Adam H. (2019). Proceedings of the IEEE/CVF international conference on computer vision.

[bib0011] Hu J., Shen L., Sun G. (2018). Proceedings of the IEEE conference on computer vision and pattern recognition.

[bib0012] Hu Y.L., Xiong J.Q., Xu J.Y., Gou Z.C., Ying Y.B., Pan J.M., Cui D. (2024). Behavior recognition of cage-free multi-broilers based on spatiotemporal feature learning. Poult. Sci..

[bib0013] Jin Z., Shu H., Hu T., Jiang C., Yan R., Qi J., Qi J.W., Guo L. (2024). Behavior classification and spatiotemporal analysis of grazing sheep using deep learning. Comput. Electron. Agric..

[bib0014] Lin C.W., Chen Z.S., Lin M.X. (2022). Video-based bird posture recognition using dual feature-rates deep fusion convolutional neural network. Ecol. Indic..

[bib0015] Lin J., Gan C., Han S. (2019). Proceedings of the IEEE/CVF international conference on computer vision.

[bib0016] Lin S.X., Pan Y.X., Huang L., Li X., Zhu Y.W. (2019). Farming and management of Lion-head goose (in Chinese). Guangdong Anim. Husband. Vet. Sci.Technol.

[bib0017] Lin W.Q., Lin S.H., Lin W.J. (2022). Characteristics of common diseases in lion-head geese and methods of prevention and treatment (in Chinese). Chin. J. Animal Husbandry Veterin. Med..

[bib0018] Liu Y.Q., Zheng Y.Z., Yang P.K., Nie L.H., Wang J.X., Shi W.B., Zhang Z.X. (2021). An overview of common diseases in Shitou geese and their prevention and treatment with traditional Chinese medicine. J. Hanshan Normal Univ..

[bib0019] Liu Y.Y., Cao X., Guo B.B., Chen H.J., Dai Z.C., Gong C.W. (2023). Research on detection algorithm about the posture of meat goose in complex scene based on improved YOLO v5. J. Nanjing Agricult. Univ..

[bib0020] Luo Y.Q., Han Y.H., Li S.C., Li X.J., Huang Y.M., Tian Y.B., Zhang X.M., Wu Z.P. (2024). Overview of germplasm resources of Shitou Goose and suggestions for protection, development and utilization. Chin. Livestock Poultry Breed..

[bib0021] Martiskainen P., Järvinen M., Skön J.P., Tiirikainen J., Kolehmainen M., Mononen J. (2009). Cow behaviour pattern recognition using a three-dimensional accelerometer and support vector machines. Appl. Anim. Behav. Sci..

[bib0023] Nasirahmadi A., Sturm B., Edwards S., Jeppsson K.H., Olsson A.C., Müller S., Hensel O. (2019). Deep learning and machine vision approaches for posture detection of individual pigs. Sensors.

[bib0024] Nasiri A., Yoder J., Zhao Y., Hawkins S., Prado M., Gan H. (2022). Pose estimation-based lameness recognition in broiler using cnn-lstm network. Comput. Electron. Agric..

[bib0025] Qi L., Bao J., Li J.H. (2009). Application of ethology in animal welfare and breeding (in Chinese). China Animal Health Inspec..

[bib0026] Selvaraju R.R., Cogswell M., Das A., Vedantam R., Parikh D., Batra D. (2017). Proceedings of the IEEE international conference on computer vision.

[bib0027] Soomro, K., Zamir, A., and Shah, M. 2012. UCF101: a dataset of 101 Human actions classes from Videos in the Wild. ArXiv, abs/1212.0402.

[bib0028] Stott A.W., Milne C.E., Goddard P.J., Waterhouse A. (2005). Projected effect of alternative management strategies on profit and animal welfare in extensive sheep production systems in Great Britain. Livest. Prod. Sci..

[bib0029] Su Q., Tang J., Zhai M., He D. (2022). An intelligent method for dairy goat tracking based on Siamese network. Comput. Electron. Agric..

[bib0030] Tran D., Wang H., Torresani L., Ray J., LeCun Y., Paluri M. (2018). Proceedings of the IEEE conference on Computer Vision and Pattern Recognition.

[bib0031] Volkmann N., Zelenka C., Devaraju A.M., Bruenger J., Stracke J., Spindler B., Kemper N., Koch R. (2022). Keypoint detection for injury identification during turkey husbandry using neural networks. Sensors.

[bib0034] Wang L., Xiong Y., Wang Z., Qiao Y., Lin D., Tang X., Van Gool L. (2016). European conference on computer vision.

[bib0036] Wang X., Girshick R., Gupta A., He K. (2018). Proceedings of the IEEE conference on computer vision and pattern recognition.

[bib0035] Wang Q., Wu B., Zhu P., Li P., Zuo W., Hu Q. (2020). Proceedings of the IEEE/CVF conference on computer vision and pattern recognition.

[bib0032] Wang C.Q., Wang Y.T., Shang S.Q., Zhang N. (2024). Research on chicken basic behavior recognition method based on YOLOv5x. Agricult. Equip. Vehicle Eng..

[bib0033] Wang J.W., Du Z., Wen B., Wu Z., Lin X. (2024). 2024 International Conference on Machine Learning and Cybernetics (ICMLC).

[bib0037] Xiao D.Q., Zeng R.L., Zhou M., Huang Y.G., Wang W.C. (2023). Monitoring the vital behavior of Magang geese raised in flocks based on DH-YoloX. Transac. Chin. Soc. Agricult. Eng. (Transac. CSAE).

[bib0038] Yang D.L., Qi J.L., Chen H., Gao Y., Wang L.Z. (2024). Individual behavioral identification and differential analysis of free-range laying hens based on improved YOLO v8n mode. Transac. Chin. Soc. Agricult. Machin.

[bib0039] Zhang Y., Sun P., Jiang Y., Yu D., Weng F., Yuan Z., Luo P., Liu W., Wang X. (2022). European conference on computer vision.

